# Roadmap to Building a Cell: An Evolutionary Approach

**DOI:** 10.3389/fbioe.2020.00927

**Published:** 2020-08-19

**Authors:** Zhanar Abil, Christophe Danelon

**Affiliations:** Department of Bionanoscience, Kavli Institute of Nanoscience, Delft University of Technology, Delft, Netherlands

**Keywords:** synthetic cell, artificial cell, liposome, bottom-up synthetic biology, directed evolution, system’s level evolution

## Abstract

Laboratory synthesis of an elementary biological cell from isolated components may aid in understanding of the fundamental principles of life and will provide a platform for a range of bioengineering and medical applications. In essence, building a cell consists in the integration of cellular modules into system’s level functionalities satisfying *a* definition of life. To achieve this goal, we propose in this perspective to undertake a semi-rational, system’s level evolutionary approach. The strategy would require iterative cycles of genetic integration of functional modules, diversification of hereditary information, compartmentalized gene expression, selection/screening, and possibly, assistance from open-ended evolution. We explore the underlying challenges to each of these steps and discuss possible solutions toward the bottom-up construction of an artificial living cell.

## Introduction

All known life forms are composed of cells as the elementary unit. Nevertheless, the staggering complexity of even the simplest known cells is prohibitive to our understanding of the most basic principles of life. Building a biological cell from scratch, i.e., from a minimal set of loose components, such as *in vitro* synthesized or purified biomolecules, would serve as a forward engineering approach, thus illuminating the design principles of life (Elowitz and Lim, 2010). Such construction of a minimal, or synthetic, cell bottom-up is considered today to be one of the grand challenges in synthetic biology. Although the challenge first arose as an approach to answering origin of life questions, the field grew considerably in recent decades, and is now also tapping into the perspectives for improving applications in biotechnology and biomedicine (Ausländer et al., 2017). Approaches that have been used so far toward building synthetic cells and their potential applications have been extensively reviewed elsewhere (Forster and Church, 2006; Luisi et al., 2006; Noireaux et al., 2011; Nourian et al., 2014; Xu et al., 2016; Jia et al., 2017; Sikkema et al., 2019; Stano, 2019). Most studies attempt to define the molecular hardware, i.e., enumerate relevant genes, proteins, biological mechanisms, and metabolic pathways that may compose a minimal cell. Yet, how practically the myriad of components will assemble into a functional cell remains a largely unexplored area. This perspective addresses this challenge by conceptualizing an evolutionary synthetic biology route. Specifically, we argue that *in vitro* evolution must be employed as an engineering tool to accelerate the optimization of individual modules as well as their integration into system’s level functionalities. Numerous iterations of module integration, genetic diversification, and phenotype assessment would gradually increase the complexity and autonomy of the system, i.e., its degree of “aliveness.”

## More Than the Sum of Its Parts

**FIGURE 1:**
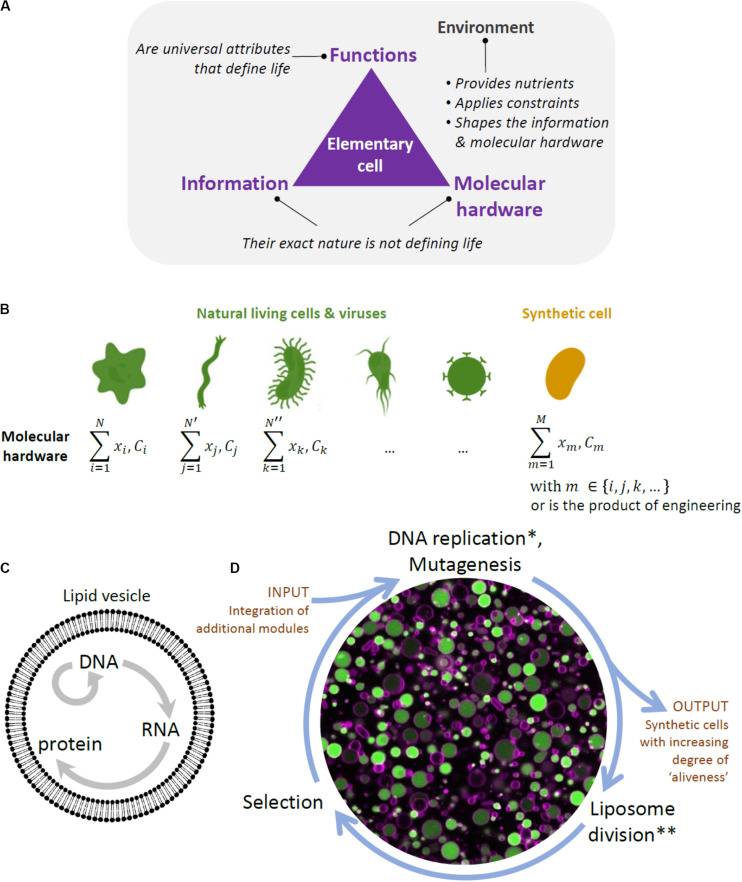
Framework to designing a synthetic cell. **(A)** Building a cell is also a way to question the fundamental principles of life. The diagram depicts our view of the key ingredients constituting a biological cell, and by extension an elementary cell. Cellular unit and environment are entangled and they both constitute the “living system”. We consider information (i.e., the sequence of nucleotides in the DNA and RNA of present-day organisms and viruses) and molecular hardware (i.e., the memory-carrying molecule and the set of proteins, lipids, cofactors, etc, composing the cell) as defining elements of life, but their exact nature is *not*, as they are both subject to diversity across organisms. In contrast, minimal cellular life can be seen as a set of universal functions that are common to all living things. **(B)** The molecular hardware is defined as the nature and concentration (*C*) of each cell’s constituents (*x* refers to their localization). It differs from one organism to the other and, even, in the modern view of biology, between individual cells from a clonal population. The molecular hardware of a synthetic cell may be a combination of natural parts derived from existing organisms and viruses, and engineering products through *in vitro* evolution or *de novo* design. The complexity (or simplicity) of the molecular hardware depends on the composition of the environment. Synthesizing a cell following this approach will yield never-born cells, whose initial conditions supporting life do not exist in nature. **(C)** Liposome and the key reactions forming the central dogma in biology. **(D)** Scheme of the *in vitro* continuous evolution cycle applied to synthetic lipid vesicles. Center, microscopy image of liposomes (colored in magenta) expressing the protein GFP (green) from its gene. *In discontinuous evolution mode, this step would consist of DNA isolation and bulk amplification. **In discontinuous evolution mode, the DNA amplified in bulk is re-encapsulated in new liposomes.

A conceptual difficulty in constructing a synthetic cell bottom-up lies in the recognition that life is not simply a construct composed of a mixture of chemicals that possesses certain physical properties, but rather is a highly complex physico-chemical phenomenon with yet to be fully understood emergent properties. In other words, to construct a living system from scratch, it is not enough to simply emulate some of life’s properties, such as compartmentalization, growth, or information replication, but bring all the processes and functions together into a phenomenon that cannot be reduced to its parts (Figures 1A,B).

One of the features that are considered to be defining of life is the functional integration of elementary processes into system’s level properties. Some of life’s functionalities, such as self-organization, self-maintenance, and information continuity, have been extensively studied and attempts have been made to at least partially reproduce them in the laboratory (Caschera and Noireaux, 2014). However, *integration* of these functionalities in a single synthetic cell still remains far out of reach (Caschera and Noireaux, 2014). Another, related, property of living systems and a goal to achieve in the efforts of building a minimal cell is *autonomy*. By autonomy, we understand a property of a far-from equilibrium, thermodynamically open system that enables it to establish an organizational identity of its own as a result of a set of endergonic-exergonic couplings of internal processes (Ruiz-Mirazo et al., 2004). Hence, a system would be considered autonomous if it is able to maintain its far-from equilibrium state by means of intrinsically governed building of its components and operation of vital processes, provided there is an inflow of necessary substrates and outflow of byproducts. Finally, while basic autonomous systems might be capable of some degree of adaptability to changing environmental conditions, this might not be enough for the survival of a cellular population in a less stable environment. Thus, there is a belief among a number of experts that mechanisms for reliable information continuity and hereditary adaptability, or *evolution*, are required for the survival of a minimal cell in the long run, and constitute one of the defining features of life (Joyce, 1994; Koshland, 2002; Ruiz-Mirazo et al., 2012). Thus, by aiming to construct a minimal cell, we are seeking to construct a functionally integrated phenomenon capable of novel, *system’s level functionalities*, such as autonomy and evolvability.

Another, practical, difficulty in building a minimal cell is the sheer volume of the variety of molecules, modules, functions, and their inter-relationship that needs to be explored. The first challenge is that, once chosen, the individual modules might not work as intended in the new environment (altered expression, degradation, loss of activities in the new environment, and broken regulation). This is particularly expected if the constituents originate from different organisms (Figure 1B). Additional engineering of individual parts would thus be necessary. Furthermore, a synthetic cell will require a functional integration of multiple modules, which implies further engineering of functional orthogonality, compatibility, and cross-regulation of parts. This approach traditionally involves detailed analysis of individual modules and laborious fine-tuning processes to make the genetic circuits, pathways, and other complex networks to function as desired. However, with increased complexity, the task becomes prohibitively difficult.

## A System’s Level Evolutionary Approach

We propose to address these difficulties of building a synthetic cell by using a system’s level evolutionary approach (Figures 1C,D), which offers an achievable alternative to the traditional rational engineering strategy. We know this thanks to the substantial contribution, in the last few decades, of directed evolution methods to engineering of a vast number and variety of proteins (Arnold, 2019) and *in vitro* evolution methods for engineering ribozymes and aptamers (Wilson and Szostak, 1999). Specifically, successful evolution of proteins *in vitro* and especially in liposomes have been demonstrated (Matsuura and Yomo, 2006; Nishikawa et al., 2012). One of the great advantages of the evolutionary approach to engineering of complex systems is that it does not require *a priori* knowledge of structure-function relationships, and, in our case, of inter-relation of multiple components in pathways, genetic circuits, or functional modules in even larger synthetic systems. Thus, we believe that due to our very limited understanding of the nature of a minimal living system, the system’s level evolutionary approach is the only practical way we can progress toward the goal of building a synthetic cell.

The system’s level evolutionary approach likewise underlines our current understanding of the origin of the first biological cell via chemical evolution (Ruiz-Mirazo et al., 2017; Takeuchi et al., 2017). Reminiscent of how chemical evolution contributed to the stepwise increase in a protocell’s complexity and dynamic kinetic stability (Pross, 2013), we envision artificial evolution driving the gradual increase in complexity of a minimal synthetic cell to a system that has a higher degree of “aliveness.” Our strategy of building a cell thus embraces the idea of fuzzy logic that allows partial set membership rather than clear-cut definition of life with strict criteria (Bruylants et al., 2010). This is not unlike how prebiotic systems are considered in a continuum of grades of “aliveness.” Therefore, building a synthetic cell via numerous intermediates from a more primitive to a more complex autonomous system could shed light on the fundamental principles of the emergence of life.

Finally, as we mentioned above, evolution is often considered as one of life’s defining features; and thus, engineering a synthetic cell precursor for evolvability may be an attractive goal in itself. Hence, the development of a continuous *in vitro* evolution approach, which will be discussed below, will not only accelerate the engineering of a synthetic cell, but will become a synthetic cell feature that ensures increased robustness and adaptability to varying environmental conditions, and will require the seamless integration of information replication, diversification, expression, selection, and distribution among daughter synthetic cells.

In the following, we would like to discuss the challenges for successful implementation of the evolutionary approach and propose possible solutions to tackle them.

## Challenges and Possible Solutions

### Choice of Scaffold

A number of scaffolds for the bottom-up construction of a minimal cell have been explored. Membranous vesicles, emulsion droplets, and coacervates all have their own strengths and disadvantages (Buddingh’ and Van Hest, 2017), and will undoubtedly contribute to the ultimate goal conceptually and as parallel experimental platforms. However, we believe that liposomes, aqueous vesicles encapsulated by a phospholipid bilayer, may be the most promising choice in the long run (Figure 1C). First, liposomes are vesicles that resemble modern cells the most, and thus have the most potential in shedding light on the mechanisms of life as we know it. Second, they are biocompatible, meaning that they can be more easily integrated with other building blocks, which will most likely come from existing biomolecule types, such as membrane proteins and polysaccharides. Biocompatibility also entails that they could be used in combination with other currently characterized biomolecules for biotechnological or biomedical applications (i.e., bioreactors for sugar production or nucleic acid delivery).

### Integration of Modules for System’s Level Evolution

The system’s level evolution of a synthetic cell may be directed and facilitated by applying a semi-rational approach. Therein, new polypeptide domains, new genes, and entire functional modules are added intermittently throughout the evolution campaign, followed by multiple rounds of additional genetic diversification and phenotypic enrichment. Multiple modules will need to be integrated in a functional way. However, the goal is not to reconstitute an existing living organism like *Escherichia coli* or a mycoplasma, but building a novel, minimal system, composed of a variety of modules, and inspired from multiple contemporary organisms and viruses (Figure 1B).

The order of addition of functional modules and concomitant re-design of the environment will need to be carefully considered. Since we will be undertaking an evolutionary approach, the primary and central module would be the one ensuring genetic heredity. One could start with a simple hereditary system capable of self-replication, such as a DNA polymerase and its auxiliary proteins (Van Nies et al., 2018). The initial set of genes could be expressed using an *in vitro* transcription-translation system, such as PURE system (Shimizu et al., 2001). Some of the modules that would require subsequent integration are those responsible for rudimentary anabolism, catabolism, various internal and external feedback and regulatory mechanisms, as well as controlled division mechanisms. When an additional module is functionally integrated (for production of a biomolecule, for example), it would remove the need for external supply of the corresponding component or physical intervention of the researcher. This will be used as the newly added selection pressure. Hence, in general, with the growing autonomy of the synthetic cell, the complexity of the environment and the need for physical intervention should decrease.

It is important to note that by integration we do not mean simply adding the individual modules to the growing DNA chain, and applying the traditional directed evolution methods, such as genetic diversification and phenotype interrogation. A traditional approach would require total destruction of vesicles, as well as extraction and subsequent re-encapsulation of DNA. This discontinuous evolution mode may be applied in the early stages of synthetic cell evolution. However, we are not interested in simply obtaining an optimal synthetic genome. After all, being in possession of an entire *E. coli* genome does not equal to having a living organism. Therefore, we suggest to build a system capable of *in vitro* continuous evolution (Figure 1D), where hereditary material gets re-distributed between daughter cells, thus providing a physical continuity of a gene-expressing vesicle population in time. Initially, it would be enabled by a researcher’s intervention (e.g., mechanical vesicle division), and eventually, will be the result of the system’s intrinsic functions (such as self-encoded division mechanism).

### Genetic Diversification of Large Synthetic Genomes

Genetic diversification drives directed evolution, and success of the campaign heavily depends on the nature, size, and quality of the genetic library (Bratulic and Badran, 2017). For single genes or shorter DNA fragments, the commonly used approaches of genetic diversification can be used (Gillam et al., 2014). Increasing complexity of a synthetic cell, however, would necessitate diversification of an increasingly larger synthetic genome, where traditional approaches may become impractical. Herein, we will discuss strategies that may become instrumental for genetic diversification of synthetic genomes, such as assembly and recombination, random, and semi-random mutagenesis methods.

**FIGURE 2:**
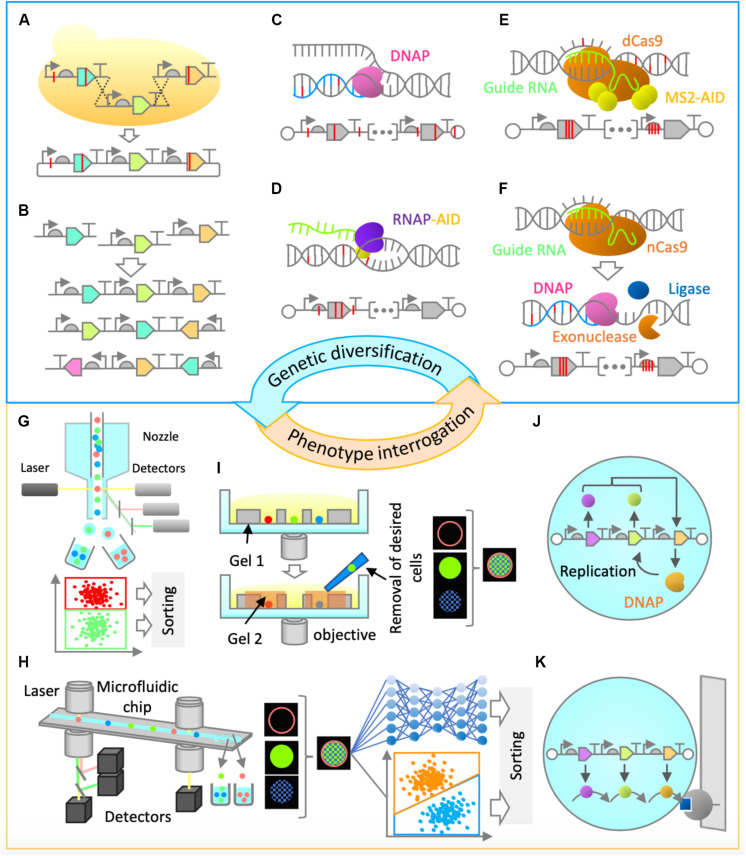
Proposed genetic diversification and phenotype interrogation methods for system’s level *in vitro* evolution of gene-expressing vesicles. **(A,B)** Combinatorial design and assembly methods **(A)** Gene assembly and recombination. Homologous recombination in yeast can be performed for gene assembly and recombination of large genetic clusters. **(B)** Gene order and orientation optimization. Constraint-based combinatorial design as well as *in vitro* assembly could be employed for gene expression optimization. **(C,D)** Random mutagenesis methods. **(C)** Low-fidelity DNA replication. Engineered Φ29 or a similar DNA polymerase can be used for error-prone replication of the entire synthetic genome, thus enabling in-vesicle random mutagenesis. **(D)** Base editing. Base editor, such as activation-induced deaminase (AID) can be fused to an RNA polymerase, enabling random mutagenesis in both strands of DNA in gene expressing regions. **(E,F)** Semi-random mutagenesis methods. **(E)** Programmable base editing. Base editors can be tethered to programmable DNA-binding proteins, which will target random mutagenesis to a narrow region of interest. For this purpose, cytidine or adenine deaminase domains (AID, APOBEC1, TadA) can be fused to MS2 coat protein and tethered to endonuclease-deficient versions of Cas9 (dCas9) or Cpf1 (dCpf1) proteins via MS2-hairpins linked to guide RNA. This flexible architecture would allow a random mutagenesis window of around 100 base pairs (Hess et al., 2016). **(F)** Error-prone *in vitro* nick repair. In this proposed method, a nick introduced by a reprogrammable nickase (such as nCas9 or nCpf1) at a region of interest is chewed back with an exonuclease (such as T5 exonuclease), filled in by error-prone DNA polymerase (such as human PolI), and ligated by DNA ligase (such as Taq DNA ligase) to resolve the nick. Such a method would likely constrain the random mutagenesis to up to 200 bases on the targeted strand. **(G–I)** Screening Methods. **(G)** Fluorescence-activated cell sorting (FACS). This high-throughput screening method relies on fluorescence measurement of individual particles and sorting by electrostatic deflection. **(H)** Intelligent image-activated cell sorting (IACS). This high-throughput screening method sorts particles based on their unique morphological features and is based on real-time image-based flow cytometry assisted by artificial intelligence. **(I)** Dual-photopolymerized microwell array sorting. This image-based single-cell sorting method relies on two-step photopolymerization process: the first to create an array of microwells to capture individual cells and the second to encapsulate undesired cells. The desired cells are removed by washing. **(J,K)** Selection methods. **(J)** Compartmentalized partnered replication. When adapted for *in vitro*, in-liposome use, this approach would rely on linking a phenotype of interest to the expression of a DNA polymerase gene. Differential amplification of a synthetic gene cluster by the expressed DNA polymerase in liposomes would be the basis of selection. **(K)** Affinity chromatography. Liposomes displaying a molecule of interest can be enriched by specific interaction with a binding partner on a solid support.

Construction of synthetic genomes will first entail the utilization of commonly used *in vitro* and/or *in vivo* genetic assembly methods (Eriksen et al., 2018; Figure 2A). The initial optimization of gene cluster architecture can be performed by combinatorial variation of part choice, gene order, gene orientation, and operon occupancy (Figure 2B), as it was shown that these factors are highly useful for genetic optimization (Smanski et al., 2014). Gene assembly methods would also enable genetic recombination, thus ensuring combinatorial rearrangement of mutations between rounds of evolution (Figure 2A).

For efficient *in vitro* diversification of large nucleic acids, one can envision the use of a replication system with high processivity, high strand-displacement activity for isothermal amplification, and low fidelity for accelerated mutagenesis. A suitable candidate with such characteristics is the Φ29 DNA polymerase, provided that its high fidelity can be reduced (Figure 2C). The fidelity of Φ29 or a similar DNA polymerase could be lowered by using increased Mn^2+^ ion concentrations (Fujii R. et al., 2014) or by utilizing exonuclease-deficient variants. Alternatively, increased mutagenesis levels could be achieved by adapting the use of DNA deaminase domains (such as cytidine or adenine deaminases) *in vitro* either alone (Al-Qaisi et al., 2018) or in fusion with T7 RNA polymerase (Chen et al., 2019; Figure 2D). Development of these and similar methods will be critical in the diversification of large synthetic genomes. However, the sequence space of a synthetic genome would be vast, and the use of random mutagenesis approaches alone may not be enough for efficient evolution campaigns.

More focused, or semi-random, mutagenesis approaches might be needed, assisted by relevant knowledge from biochemical and bioinformatical studies, or simply by modulation of relative gene expression levels in the multi-gene system. For example, to target the mutagenesis to a narrow region of interest, deaminase domains can be tethered to reprogrammable DNA-binding proteins, such as endonuclease-deficient Cas9 or Cpf1 (Eid et al., 2018; Figure 2E). The attractiveness of this approach lies in the fact that it does not require a double strand break or a complex cellular repair machinery, and it could be adapted for use *in vitro*. Alternatively, one could engineer a method whereby a nick introduced by a reprogrammable nickase at a region of interest is subsequently or simultaneously treated with an exonuclease to chew back the nicked strand, an error-prone DNA polymerase to mutagenically fill in the gap, and a ligase to covalently repair the nick (Figure 2F). This would be reminiscent of the commonly used cloning method (Gibson et al., 2009), but would not require *in vitro* assembly of multiple fragments followed by *in vivo* screening, and would be applicable for a more focused *in vitro* mutagenesis of large DNA fragments. These and other genetic diversification methods still require extensive development and implementation *in vitro*; however, the effort will be vital for successful system’s level evolution and synthetic cell construction.

### Selection/Screening Strategies

The next challenge is to develop efficient approaches to enrich a population of liposomes for synthetic cells’ desired phenotypes (Figure 1D). For certain phenotypes, traditional screening and selection methods (Packer and Liu, 2015) can be implemented. For example, if a phenotype of interest can be linked to the expression level of a single gene, such as a fluorescent or luminescent protein, high-throughput screening by fluorescence activated cell sorting (FACS) can be implemented (Fujii S. et al., 2014). Fluorescence activated cell sorting, the most common method for sorting particles, allows for high-throughput and multiparameter sorting (Figure 2G). It could also be implemented in cases where a substrate is fluorogenic or affinity-based enrichment is required. Alternatively, it is possible to exploit selection strategies by linking a phenotype to the expression of a DNA polymerase (Abil et al., 2017), given that it is adapted for *in-liposome* amplification (Figure 2J) or to a destructive protein (negative selection by destabilizing the liposomes). For affinity-based enrichment, FACS could potentially be substituted with affinity chromatography (Figure 2K). In many cases, though, traditional approaches that are suitable for evolution of single genes or simple pathways may not be appropriate enough for system’s level evolution, since there will likely be no single parameter defining the system’s state for high-throughput screening or selection. Therefore, a paradigm shift in the approaches to tackle this problem is needed.

One way of enriching vesicles for system’s level functionalities could be via imaging-based screening methods. Cells’ (and by extension, vesicles’) “physiological” functions often manifest in morphological identities and molecular localization footprints, and therefore an imaging-based screening approach would offer an unprecedented advantage over conventional high-throughput screening methods, such as FACS. One emerging technology that would enable enrichment based on multidimensional fluorescence images is intelligent image-activated cell sorting (IACS; Figure 2H; Nitta et al., 2018; Isozaki et al., 2020). Image-activated cell sorting integrates microfluidics, high-throughput cell microscopy, real-time intelligent image processing, decision making, and cell sorting. The strategy has the potential for opening the door for the exploration of phenotypes that are more directly connected to vesicles’ system’s level functions. A possible alternative to IACS is image-based sorting via dual-photopolymerized microwell arrays (Figure 2I; Sun et al., 2013). This method relies on microwell array-assisted spatial segregation, imaging and identification, and screening based on two-step photopolymerization. This approach offers the advantage of interrogation based on high-resolution imaging, as well as time-lapse and long-term dynamics, an advantage that could be uniquely suited for system’s level evolution. Adapting this methodology for screening of micron-sized vesicles and improving it by significantly increasing the throughput capacity would be desirable.

### Open Ended Evolution

As an alternative approach to the semi-rational evolutionary approach discussed above, one could choose to undertake a more exploratory, open ended, evolution. Herein, an intrinsic mechanism would be provided for the exploration of new state spaces by the system, thus allowing expansive and transformational evolutionary novelties (Banzhaf et al., 2016; Taylor, 2019). For example, new state spaces for exploration can be provided simply by modifying and re-engineering of the environment. Other mechanisms could be exploitation of exaptation, where a structure that was performing a certain function can switch to performing a different function; and compositional systems, where a new or enhanced function emerges from the combination of smaller part types. Finally, to aid in the discovery of difficult-to-identify features, one can explore the utilization of unsupervised machine learning (Lu et al., 2019). Open ended evolution could provide new unexpected solutions to evolutionary challenges. However, its requirements and mechanisms are still poorly understood and its implementation will require intensive investigation.

## Conclusion

Herein, we proposed a system’s level evolutionary approach for building a synthetic cell. We believe that it delineates the paradigm shift needed to reach this ambitious goal. A number of new methodologies still need to be developed for the implementation of this vision. However, each of these developments will not only bring us closer toward a synthetic cell, but also open the doors to the realization of novel applications in synthetic biology. Infusing cell-free biology with system’s level evolution will provide testbeds for exploring the roots of evolutionary processes in living organisms, as well as a new perspective in our understanding of life’s fundamental properties. It would also significantly contribute to the ongoing fluid interrelationship between theoretical definition of life and empirical assessment thereof (Amilburu et al., 2020).

The evolutionary strategy will still require expertise, considerable effort, and coordination of more than a single group of researchers. These efforts will be further complicated by the fact that numerous evolutionary routes will likely have to be explored to arrive at a more viable one. Therefore, to facilitate reproducibility and distribution of materials between different groups working on this unifying goal, it would be beneficial to establish or improve platforms for standard part repository (Beal et al., 2020).

A sceptic might wonder if building a synthetic cell via evolution (which encompasses a level of randomness), as opposed to rational engineering, can be useful for our efforts of understanding life and its origins. The first drawback of our approach is that the final system’s structure and performance will be highly dependent on its evolutionary history, which would be contingent on experimenter’s choices of modules and environmental design, as well as random mutagenesis and genetic drift. The second drawback is that the evolutionary path is extremely unlikely to repeat the prebiotic evolution of terrain life, mostly because of the difficulty to reconstitute in the laboratory the environmental conditions that prevailed on the early Earth. Nonetheless, if we manage to construct artificial life using *in vitro* continuous evolution, this would support the general idea that terrain life likely originated from similar evolutionary processes. If, on the other hand, evolution is found to be the *only* way of constructing a living organism, this fact alone would illuminate a novel view on the genesis of living matter from non-living as well as reveal a fundamental feature of life. The description of life as an *evolved* and *evolving* phenomenon would thus transform from a theoretical concept to an empirical observation.

## Author Contributions

Both authors contributed to conceptualization, writing, and funding acquisition.

## Conflict of Interest

The authors declare that the research was conducted in the absence of any commercial or financial relationships that could be construed as a potential conflict of interest.
